# MOTS-c Serum Concentration Positively Correlates with Lower-Body Muscle Strength and Is Not Related to Maximal Oxygen Uptake—A Preliminary Study

**DOI:** 10.3390/ijms241914951

**Published:** 2023-10-06

**Authors:** Remigiusz Domin, Michał Pytka, Mikołaj Żołyński, Jan Niziński, Marcin Rucinski, Przemysław Guzik, Jacek Zieliński, Marek Ruchała

**Affiliations:** 1 Department of Endocrinology, Metabolism and Internal Medicine, Poznan University of Medical Sciences, 60-355 Poznan, Poland; mruchala@ump.edu.pl; 2 University Centre for Sport and Medical Studies, Poznan University of Medical Sciences, 60-802 Poznan, Poland; mjpytka@gmail.com (M.P.); mik.zolynski@gmail.com (M.Ż.); jan.nizinski@gmail.com (J.N.); pguzik@ptkardio.pl (P.G.); 3 Department of Cardiology, Intensive Therapy, Poznan University of Medical Sciences, 60-355 Poznan, Poland; 4 Department of Histology and Embriology, Poznan University of Medical Sciences, 60-355 Poznan, Poland; 5 Department of Athletics, Strength and Conditioning, Poznan University of Physical Education, 61-871 Poznan, Poland; jacekzielinski@wp.pl

**Keywords:** mitochondria-derived peptide, muscle power, muscle force, exercise, countermovement jump test, exercise physiology

## Abstract

The mitochondrial open reading frame of 12S rRNA-c (MOTS-c) is a mitochondrial-derived peptide that regulates the nuclear genome during stressful conditions such as hypoxia, which is typical of exercise and training. We aim to mainly investigate the relationship between serum MOTS-c concentration and muscle strength parameters measured during the countermovement jump test with oxygen consumption (VO2) measured during the cardiopulmonary exercise test to exhaustion. Physically active healthy volunteers (17 male, three female, median age 30 years), not involved in any regular exercise program or participating in any sports competitions, performed five consecutive countermovement jump tests and cardiopulmonary exercise tests until maximal exhaustion and underwent a body composition assessment by means of bioelectrical impedance analysis, and had serum MOTS-c concentration measured at rest. Serum MOTS-c concentration was positively correlated with the average power and average and maximal force of the jumps, both overall muscle mass and leg muscle mass, but not with body fat percentage. There was no correlation with peak VO2. A higher serum MOTS-c concentration is associated with greater muscle mass, force, and power generated during jumping in healthy individuals but not exercise capacity reflected by peak VO2. More studies are needed to better understand the physiological and clinical values of these findings and why MOTS-c is better associated with measures of muscle strength and not endurance in physically active people.

## 1. Introduction

Muscle strength and power generation depend on structural and biochemical adaptations to exercise. These adaptations include macro- and microstructural muscle changes with motoneuronal recruitment, metabolic alterations, an increased number and size of mitochondria, and adenosine triphosphate (ATP) content. During high-intensity physical activity such as the countermovement jump (CMJ) test, contracting muscles require large amounts of immediate energy supply from ATP and phosphocreatine turnover [1]. 

In response to physical activity, contracting muscles and other tissues release exercise-induced cytokines (e.g., myokines, adipokines, hepatokines). These molecules act as hormones with autocrine, paracrine, or endocrine activity [2]. 

Mitochondria-derived peptides (MDPs) are small and comprise fewer than 100–150 amino acids encoded by mitochondrial DNA (mtDNA). They facilitate communication between mitochondria and the nucleus. MDP blood concentration increases due to exercise and is considered a myokine [3,4]. Humanin and small humanin-like peptides 1-6 (SHLP1-6) were the first described MDPs [5]. In 2015, Lee et al. discovered a new MDP—the mitochondrial open reading frame of the 12S rRNA-c (MOTS-c)—a 16-amino-acid peptide targeting the muscles and regulating their metabolism [6]. 

MOTS-c originates in the mitochondria but is translated and synthesised in the cytoplasm. This protein regulates energy metabolism, including glycolysis, muscle glucose uptake, and beta-oxidation, primarily through the folate-AICAR-AMPK pathway. It inhibits the folate cycle, which results in the accumulation of AICAR (aminoimidazole-4-carboxamide): a potent AMP-activated protein kinase (AMPK) activator. In this way, MOTS-c affects energy metabolism, insulin resistance, inflammatory response, exercise, and ageing [6,7]. 

Under metabolic stress (e.g., glucose deprivation or oxidative stress during exercise), MOTS-c is translocated to the nucleus in an AMPK-dependent mechanism. This process remains poorly understood. MOTS-c controls nuclear gene expression in the nucleus, particularly the antioxidant response element, which is a part of cellular stress resistance. When exposed to glucose restriction, cells overexpressing MOTS-c have better survival. MOTS-c activates metabolic pathways that maintain energy homeostasis and prevent cell apoptosis [6,8]. Due to its systemic action, MOTS-c can be qualified as a hormone [9]. 

MOTS-c activity has been also associated with the increased expression of genes involved in skeletal muscle mitochondrial biogenesis and function, leading to improved exercise capacity and endurance [10]. In addition, MOTS-c improves glucose metabolism and insulin sensitivity are both essential for muscle performance [6,7]. 

**Figure 1:**
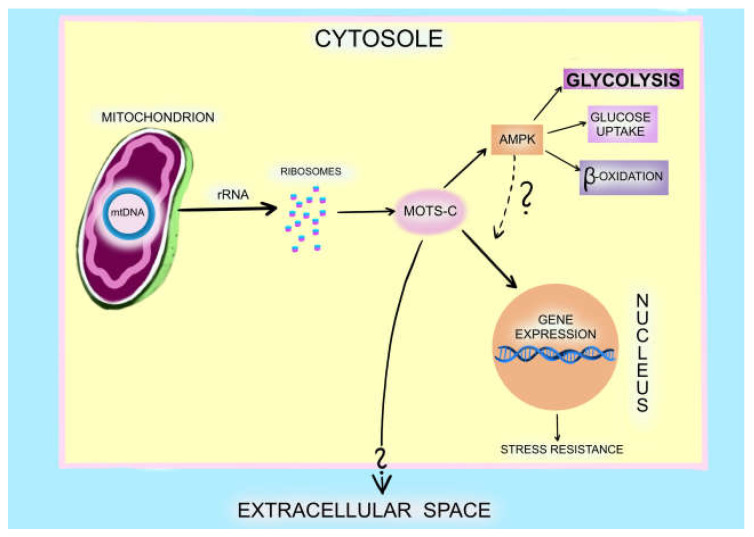
Mechanisms of MOTS-c metabolism regulation.

MOTS-c is expressed in various tissues and is present in circulation. Skeletal muscle and adipose tissue are the main targets of MOTS-c activity [11]. Figure 1 presents the known and proposed mechanisms of MOTS-c, regulating cellular metabolism and homeostasis.

MOTS-c is encoded by mitochondrial DNA (mtDNA). Its rRNA transcript (hence its name: mitochondrial open reading frame of 12S rRNA-c) is transferred outside of the mitochondria to ribosomes in the cytosol, where it is translated into MOTS-c. In the cytosol, MOTS-c regulates the activity of AMPK in anaerobic glycolysis, with glucose uptake from the extracellular space and beta-oxidation in the mitochondria. AMPK is responsible for the translocation of MOTS-c to the nucleus during metabolic stress. In the nucleus, MOTS-c regulates adaptive gene expression, promoting the development of cellular resistance to stress. It is unknown as to how MOTS-c is translocated to extracellular space.

Most data on MOTS-c and energy metabolism come from animal studies. In dystrophic mice, exogenous MOTS-c (intravenous injection) improved muscle function by increasing glycolysis and ATP concentration [12]. Recently, Hyatt et al. showed that even a single dose of exogenous MOTS-c improved exercise capacity in mice [13]. Reynolds et al. reported similar observations but with different doses of MOTS-c [10]. More on the metabolic and regulatory effects of MOTS-c can be found elsewhere [9].

Studies on MOTS-c and exercise parameters in humans are limited. We investigated the correlation between serum MOTS-c concentration and body composition alongside measures of explosive (muscle strength and power parameters) and endurance (peak oxygen consumption, VO2_max_) exercise in healthy, non-sedentary people who are physically active but do not participate in any sports competition. 

## 2. Results

### 2.1. Study Group Characteristics

The median age of all participants was 30 years (LQ-UQ: 25.75–32.25 (lower to upper quartiles)), and the majority of participants, 17 (85%), were men. Table 1 summarises the remaining clinical characteristics.

### 2.2. MOTS-c Serum Concentration, Muscle Strength Parameters, Muscle Mass, and Oxygen Uptake

**Figure 2:**
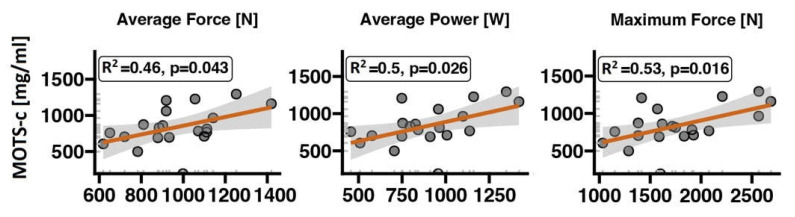
Significant associations of MOTS-c serum concentration and countermovement jump test parameters. The red line represents the regression line and the grey bands 95% confidence interval.

**Figure 3:**
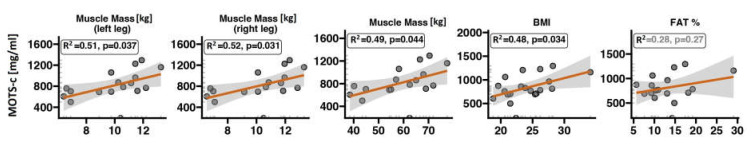
Associations of MOTS-c serum concentration, muscle mass, BMI and FAT%. The red line represents the regression line and the grey bands 95% confidence interval.

**Figure 4:**
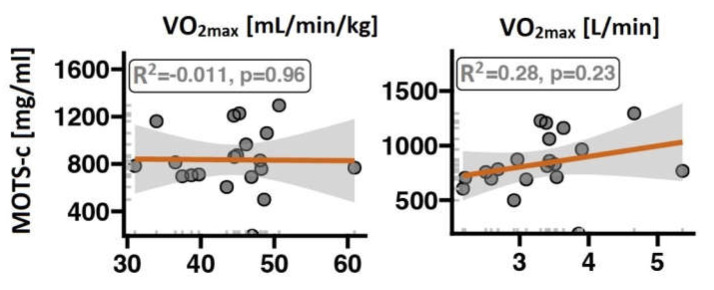
Associations between MOTS-c concentration and VO2_max_ or VO2_max_ normalised to body mass. The red line represents the regression line, and the grey band is its 95% confidence interval.

The MOTS-c serum concentration is significantly and positively correlated with indices of lower-body muscle strength during a CMJ (Figure 2). Volunteers with higher MOTS-c had a higher average and maximal force and average power. A higher MOTS-c concentration was also positively correlated with muscle mass and BMI but not with FAT% (Figure 3). There was also no correlation between MOTS-c concentration and VO2_max_ after normalisation to body weight (Figure 4).

## 3. Discussion

We found positive correlations between serum MOTS-c concentration and lower-body strength parameters such as muscle force and power during a CMJ in a group of young, healthy, and physically active subjects not involved in sports competitions. Furthermore, MOTS-c was significantly associated with muscle mass and BMI. MOTS-c was not correlated with VO2_max_ during the CPET to exhaustion or FAT%.

Exercise-related cytokines (i.e., MDPs) mediate the cross-talk between skeletal muscle and other organs and regulate whole-body metabolism [14]. MOTS-c belongs to the MDPs, and it has been considered a promising target as an exercise mimetic or physical performance enhancer [4].

The effect of acute and chronic exercise on MOTS-c has been investigated in a few studies. Von Walden et al. found that an acute bout of endurance, but not resistance, exercise increased plasma MOTS-c concentration 30 min and 3 h after exercise. Its muscle content was not affected by either type of exercise in 10 humans. The endurance exercise consisted of 45 min of cycling at 70% for the estimated VO2_max_, while the resistance exercise consisted of four sets of seven maximum repetitions of leg press and knee extensions. There was no correlation between VO2_max_ or leg strength [15].

Reynolds et al. found that the plasma MOTS-c concentration increased approximately 1.5-fold immediately after exercise (10 intervals of 60 s of cycling at VO2_max_ followed by 75 s of rest/low-intensity cycling) and returned to the baseline 4 h later. Interestingly, endogenous MOTS-c muscle content was also increased almost 12-fold after exercise and remained elevated 4 h after exercise, in contrast to the findings of Walden et al. [10]. Varying exercise intensities may explain the differences in MOTS-c muscle content between the studies conducted by Reynolds et al. and Walden et al.

Alser et al. investigated the effect of chronic endurance exercise on MOTS-c levels in professional athletes (low/moderate endurance: *n* = 47; high endurance: *n* = 28) and sedentary controls (*n* = 30). A chronic endurance exercise was associated with a lower serum MOTS-c concentration compared to the control group, and even more so in high endurance athletes (e.g., cycling, triathlon) than in low/moderate endurance athletes (e.g., football) [16]. They speculated that a lower MOTS-c concentration may reflect a mechanism of adaptation to chronic exercise in professional endurance athletes. 

In our study, the subjects were considered to be physically active, but none participated in sports competitions. They were leisure athletes with more or less regular endurance training. MOTS-c correlated with the muscle strength parameters, which are better fitted to reflect explosive/strength sports. Additionally, MOTS-c was associated with muscle mass, which is usually lower in endurance sports (i.e., marathon runners) but higher in explosive/power sports (i.e., sprinters or powerlifters).

Contrarily, we found that MOTS did not correlate with VO2_max_, which is often used to approximate exercise capacity in endurance sports. No detailed information on the participants in the study by Alser et al. makes it difficult to compare with our observations.

### 3.1. MOTS-c, Muscle Strength, and Type of Fibres

Typically, muscle mass is associated with higher muscle strength and power. In our study, higher MOTS-c concentrations were positively correlated with both muscle mass and muscle strength/power indices. 

Muscle strength and power generation are multifactorial processes that depend on the type and number of muscle fibres recruited. Fast-twitch muscle fibres (type IIx and type IIa) are responsible for dynamic and explosive exercise. They produce stronger and faster bursts of power in comparison to slow-twitch (type I) muscle fibres. The association between MOTS-c and muscle strength/power generated during an explosive effort suggests that an increased proportion of fast-twitch muscle fibres is related to MOTS-c. An indirect confirmation comes from the study conducted by D’Souza et al., who found that with an age-related progressive shift from fast to slow (type II to type I) muscle fibres, plasma MOTS-c decreases, while its muscle content increases [17]. D’Souza et al. suggested that this process is caused by the age/fibre shift compensatory mechanism or the loss of the ability of MOTS-c to exit the cell in ageing muscle. If this is the case, then subjects with a higher serum but not muscle MOTS-c concentration should have a greater proportion of type II muscle fibres and a better ability to generate force and power, as in a CMJ. This hypothesis could explain our findings. Unfortunately, we do not have the measures of MOTS-c from biopsy muscles, and thus, we cannot confirm this speculation. 

Recently, Kumagai et al. showed that MOTS-c acts as a myostatin inhibitor, a very potent inhibitor of muscle hypertrophy and differentiation. It prevents muscle fibre atrophy in rats fed a high-fat diet. MOTS-c and myostatin blood concentration are inversely correlated in humans [18]. Based on these observations, we hypothesise that MOTS-c may have some muscle-mass-enhancing properties in humans and the generation of higher power during a CMJ. Moreover, it is possible that MOTS-c may play a role in the development of muscle strength independent of muscle mass. Further research is needed to determine the exact relationship between MOTS-c, muscle mass, and muscle strength.

### 3.2. MOTS-c/ATP Muscle Strength Hypothesis 

**Figure 5:**
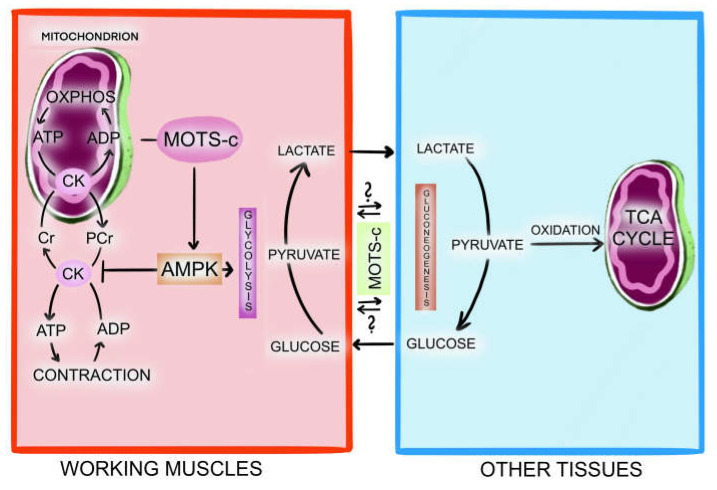
The hypothesis that MOTS-c facilitates crosstalk between muscle and other organs/tissues. We speculate that MOTS-c present in the blood is involved in inter-organ communication to maintain energy balance, probably as part of the lactate shuttle between working muscles and non-working muscles/organs involved in lactate metabolism and/or gluconeogenesis. The concentration of MOTS-c in the blood could indicate the degree to which various organs are willing to communicate with each other to maintain energy balance. Rapidly contracting muscles utilise glucose through glycolysis to generate ATP and lactate. Later, lactate can directly or indirectly enter the mitochondria after becoming pyruvate. When excessive, lactate is immediately released from the active muscle cell into neighbouring cells and the circulation. To prevent acidosis, lactate is eliminated from the bloodstream by cardiomyocytes, liver, kidneys, and other skeletal muscle cells. It is either oxidized in the mitochondria to produce ATP or converted into glucose through gluconeogenesis. The newly synthesised glucose is then released back into the circulation and taken up by working muscles. During the early stage of muscle contraction, myocytes utilise the energy stored in the phosphagen system, which is regulated by creatine kinase in the mitochondria and cytosol. Creatine kinase utilises stored phosphocreatine to synthesise ATP from ADP. Nonetheless, phosphocreatine amounts are restricted. When the stores of phosphocreatine are depleted, ATP resynthesis primarily relies on glycolysis. AMPK regulates the glycolysis rate and creatine kinase activity (enhancing the glycolysis rate while inhibiting creatine kinase). We propose that by regulating AMPK activity, MOTS-c may have a function in regulating glycolysis and ATP-phosphocreatine turnover—a vital energy source in contracting muscles.

Muscle force and power reflect skeletal muscle strength depending on the stored ATP and phosphocreatine. Phosphocreatine is a high-energy phosphate compound that is synthesised in the mitochondria and transported to the cytoplasm to serve as a temporal energy buffer and to regenerate ATP from ADP with creatine kinase. Pooled phosphocreatine in the cytoplasm can donate a phosphate group to ADP to form ATP only during the initial 8–10 s of maximal muscular effort, like a CMJ [19]. MOTS-C correlates with muscle strength indices and has been found to increase exercise performance in mice by increasing glycolysis rate and ATP concentration. [12] Considering all these data, we propose a hypothetical mechanism of crosstalk between muscle and other organs/tissues involving MOTS-c to explain our observation. (Figure 5). 

### 3.3. Limitations

A small sample size, high intrasubject results variation, an unequal gender contribution, lack of control group, and the homogeneity of race (Poles represent European ethnicity) are key study limitations. We studied volunteers aged 23–47 years; therefore, the results may not be applicable to people outside of this age range. However, this study was initiated as a proof-of-concept post hoc analysis of already available blood samples and CMJ and CPET results. We intend to use these preliminary results as the basis for a larger prospective project. 

The reported mean values of endogenous MOTS-c serum/plasma concentrations vary widely in the literature. Knoop et al. established reference values for MOTS-c plasma concentration in healthy humans of 45.9–218.5 ng/mL (10 men, median age 36 years; 10 women, median age 31 years). The mean MOTS-c serum concentration in our study was several times higher than in the population studied by Knoop et al. In our study, MOTS-c was measured by ELISA in serum samples, whereas in other studies, it has been measured in serum or plasma in different ways [20]. The small sample size prevents us from building reliable bivariate regression models that include muscle mass, muscle strength indices, and MOTS-s concentration. Therefore, we cannot determine whether MOTS-c might affect muscle strength and force generation independent of muscle mass. Future research should be conducted in larger groups of different races and ages, comparing differences between male and female participants with different levels of physical activity (i.e., sedentary controls vs. endurance/strength athletes). The CMJ focuses mainly on lower-body muscles; thus, the relationship between MOTS-c and the upper body should also be investigated.

## 4. Materials and Methods

### 4.1. Bioethical Issues

All data were collected, managed, and stored confidentially using the Redcap data collection tool hosted at the Poznan University of Medical Sciences (https://redcap.ump.edu.pl). All data were kept anonymous during data storage and analysis. Written informed consent was obtained from all subjects prior to enrolment. 

### 4.2. Study Group

The study group was based on a post hoc analysis of data collected in other studies. Twenty healthy volunteers of Caucasian ethnicity with a median age of 30 years, including 17 males and 3 females, were recruited to participate in this study. To qualify, participants had to be between 18 and 50 years old, have no history of chronic disease, and do at least one hour of regular physical activity per week. None of them had participated in structured exercise or sports competitions. Exclusion criteria included any chronic disease based on medical history and the regular use of substances on the World Anti-Doping Agency (WADA) list. Medical history was assessed by an experienced physician.

Participants abstained from strenuous exercise and alcohol for at least 24 h and from caffeinated beverages/supplements for at least 12 h prior to testing. They were asked to report to the laboratory in a postprandial state approximately 2 h after their last meal.

### 4.3. Measurements

Each subject was examined by a physician. Anthropometric parameters, including an approximation of muscle mass and FAT%, were measured using a stadiometer and four-frequency total body impedance for body composition analysis (Tanita MC 180 MA, Tanita, Tokyo, Japan).

### 4.4. Countermovement Jump Test

Maximal force and power generation were tested by performing five consecutive CMJs with a short recovery period (30 s) between jumps on the HUR Force Platform FP4 (HUR Labs, Kokkola, Finland) according to the standard operating procedures proposed by Petrigna et al. [21]. Briefly, during the CMJ test, the subjects stood on the platform with their hands on hips and performed a maximal vertical jump. The results regarding muscle power and force were averaged over the five jumps. The average force and power during a jump were calculated as the average of the entire concentric phase (extension of the hips, knees, and ankles) of the jump, and the maximum was the largest value of the 15 ms intervals during the concentric phase.

### 4.5. Peak Oxygen Uptake (VO2_max_)

The peak oxygen uptake measured during CPET to exhaustion was considered to be the maximal value. Although peak VO2 corresponds to VO2_max_ in some people, it is frequently considered to be the maximal VO2, at least during the completed CPET. CPET was performed on a specialised cycle ergometer (Excalibur Sport 2, Lode, Groningen, The Netherlands) using a CPET system (Vyntus CPX, Vyaire Medical, Mettawa, IL, USA) with gas analysers determining breath-by-breath oxygen (O_2_) and carbon dioxide (CO_2_) concentrations in inspired and expired air. The CPET system was calibrated following the manufacturer’s recommendations before each test. CPET was performed to exhaustion using an individualised incremental ramp protocol. Each CPET began with resting recordings, followed by a three-minute warm-up. After the progressive exercise started, it continued until the participants’ exhaustion or until clinically indicated (e.g., due to angina or dyspnea).

### 4.6. Blood Sampling, Storage, and Biochemical Analysis of MOTS-c

Peripheral venous blood was collected from the subjects 5 min before the CMJ test, centrifuged to obtain the serum, and stored at −80 degrees Celsius. MOTS-c serum concentration was estimated using the ELISA method by means of the MOTS-c ELISA kit (Cloud-Clone Corp Cat# CEX132Hu, RRID:AB_2933989). Each assay was performed in duplicate and the mean of the results was calculated.

### 4.7. Statistical Analysis

The normality of data distribution was tested using a graphical approach (Q–Q plots) as well as the D’Agostino–Pearson test. Data meeting the assumptions of a normal distribution were summarised as the mean with a standard deviation. Data with a non-Gaussian distribution are presented as a median with an interquartile range. Pearson correlation with an r-coefficient value and linear regression were used to examine the associations between serum MOTS-c concentration and CMJ outcomes. Only *p* < 0.05 was considered to be significant. All statistical analyses were performed in the R programming language and development environment, supported by the ggplot2 graphing library.

## 5. Conclusions

We show that MOTS-c concentration is positively correlated with muscle mass and lower-body muscle strength parameters in the CMJ test but not VO2_max_ in relation to muscle endurance. Further research is needed to fully understand the relationship between MOTS-c and muscle structure and function.

## Figures and Tables

**Table 1:** Clinical characteristics of the study group. *n* = 20, 17 male and 3 female. Muscle strength parameters were obtained from five consecutive measurements. VO2_max_ was assessed with a cardiopulmonary exercise test performed to exhaustion.

Parameter	Mean	SD
BMI	24.14	3.68
Muscle Mass [kg]	59.09	11.7
FAT%	13.32	5.54
MOTS-c [ng/mL]	834.91	265.85
Maximum Power [W]	3398.77	1044.92
Maximum Force [N]	1763.45	467.52
Average Power [W]	896.39	255.18
Average Force [N]	964.12	200.26
Peak Oxygen Uptake (VO2_max_) [L/min]	3.33	0.77

Abbreviations: SD—standard deviation; BMI—body mass index; FAT%—body fat percentage; MOTS-c—mitochondrial open reading frame of the 12S rRNA-c; W—Watts; N—Newtons.

## Data Availability

The data that support the findings of this study are available from the corresponding author upon reasonable request.
